# Morphological characteristics of the posterior malleolar fragment according to ankle fracture patterns: a computed tomography-based study

**DOI:** 10.1186/s12891-018-1974-1

**Published:** 2018-02-13

**Authors:** Young Yi, Dong-Il Chun, Sung Hun Won, Suyeon Park, Sanghyeon Lee, Jaeho Cho

**Affiliations:** 1 Seoul Foot and Ankle Center, Inje University Seoul Paik Hospital, Seoul, Republic of Korea; 2 Department of Orthopedic Surgery, Seoul Hospital, Soonchunhyang University College of Medicine, Seoul, Republic of Korea; 3 Department of Biostatistics, Seoul Hospital, Soonchunhyang University College of Medicine, Seoul, Republic of Korea; 4 Department of Orthopaedic Surgery, Chuncheon Sacred Heart Hospital, Hallym University, 77, Sakju-ro, Chuncheoni-si, Gangwon-do 200-704 Republic of Korea

**Keywords:** Ankle fracture, Computed tomography, Posterior malleolar fragment, Morphology

## Abstract

**Background:**

The posterior malleolar fragment (PMF) of an ankle fracture can have various shapes depending on the injury mechanism. The purpose of this study was to evaluate the morphological characteristics of the PMF according to the ankle fracture pattern described in the Lauge-Hansen classification by using computed tomography (CT) images.

**Methods:**

We retrospectively analyzed CT data of 107 patients (107 ankles) who underwent surgery for trimalleolar fracture from January 2012 to December 2014. The patients were divided into two groups: 76 ankles in the supination-external rotation (SER) stage IV group and 31 ankles in the pronation-external rotation (PER) stage IV group. The PMF type of the two groups was assessed using the Haraguchi and Jan Bartonicek classification. The cross angle (α), fragment length ratio (FLR), fragment area ratio (FAR), sagittal angle (θ), and fragment height (FH) were measured to assess the morphological characteristics of the PMF.

**Results:**

The PMF in the SER group mainly had a posterolateral shape, whereas that in the PER group mainly had a posteromedial two-part shape or a large posterolateral triangular shape (*P* = 0.02). The average cross angle was not significantly different between the two groups (SER group = 19.4°, PER group = 17.6°). The mean FLR and FH were significantly larger in the PER group than in the SER group (*P* = 0.024, *P* = 0.006). The mean fragment sagittal angle in the PER group was significantly smaller than that in the SER group (*P* = 0.017).

**Conclusions:**

With regard to the articular involvement, volume, and vertical nature, the SER-type fracture tends to have a smaller fragment due to the rotational force, whereas the PER-type fracture tends to have a larger fragment due to the combination of rotational and axial forces.

## Background

Ankle fractures commonly occur with an overall age- and sex-adjusted incidence rate of 187 per 100,000 person-years [1]. Trimalleolar fracture with the posterior malleolar fragment (PMF) accounted for 7% of ankle fractures [2]. Clinical studies have shown that the presence of a posterior malleolar fragment (PMF) is important as a prognostic factor or functional outcome in the treatment of ankle fractures [3–6]. Although fixation is recommended for fragments involving 25–30% of articular surface based on the articular involvement of the overall trimalleolar fracture, technical treatment strategies, such as indication or method for fixation of the PMF, remain controversial [7–10].

To understand the pathoanatomy or morphology of the PMF, some studies have been undertaken using plain radiographs [11–13], computed tomography (CT) cross-section [14–16], and 3-D reconstruction CT [17, 18]. However, most studies had proven that using plain radiographs are inadequate to properly understand the pathoanatomy of this PMF. In a few studies that examined PMFs using CT, the authors concluded that PMFs resulted from ankle and pilon fractures. To date, two classification systems [14, 17] of assessing PMF morphology of ankle fractures have been addressed through CT-based studies. Despite the fact that these two classification systems [14, 17] were able to describe the pathoanatomy or morphology of the PMF in ankle fracture, neither classification described the morphological characteristics of the PMF according to the ankle fracture pattern or injury mechanism. Therefore, a better understanding of morphological characteristics of the PMF depending on the mechanism of the injury could be useful to surgeon on planning the fixation of the PMF and may provide a basis for prognosis.

The most commonly used classification systems of ankle fractures are the Lauge-Hansen and Dannis-Weber/AO classification systems. The Dannis-Weber/AO classification system is simple to understand due to the coordinating role of the fibula and syndesmosis of the ankle joint. On the other hand, the Lauge-Hansen classification system is complex and cumbersome due to the need to understand the different stages of pathological damage in addition to the fracture pattern depending on the injury mechanism. For these reasons, the Dannis-Weber/AO classification system has more reliability and reproducibility compared to the Lauge-Hansen classification system [19]. Despite these shortcomings, the Lauge-Hansen system provided the most clinically relevant information, because the ankle fractures are categorized as basis for injury mechanism using a combination of foot position and direction force [1].

The aim of the present study was to evaluate the morphological characteristics of the posterior malleolar fragment (PMF) according to the ankle fracture pattern described in the Lauge-Hansen classification on the basis of a comprehensive CT.

## Methods

We retrospectively reviewed consecutive patients who underwent surgery for ankle fracture from January 2012 to December 2014. The following are the exclusion criteria in this study: patients who did not undergo a CT exam before surgery; had fractures without a PMF; were below 18 years old; had previous deformity; and had a significant vertical shift or comminuted PMF, which was unavailable for measurement. We retrospectively analyzed CT data of 107 patients (107 ankles) who underwent surgery for trimalleolar fracture including PMF. Two orthopedic surgeons (JC; orthopedic surgeons, foot and ankle expert, 9 years of experience and SL; orthopedic resident, trauma semi-expert, 3 years of experience) divided the patients into the following groups (according to the Lauge-Hansen classification): 76 ankles in the supination-external rotation (SER) stage IV group and 31 ankles in the pronation-external rotation (PER) stage IV group.

To assess the PMF morphological characteristics of the ankle fractures of each group according to the abovementioned classification systems, all cases from both groups were respectively categorized by the same two orthopedic surgeons using the Haraguchi [14] and Jan Bartonicek [17] classification. Based on the Haraguchi classification, the cases were categorized into three types: type I, the posterolateral-oblique type; type II, the medial-extension type; and type III, the small-shell type. Based on the Jan Bartonicek classification, the cases were categorized into five types: type 1, the extraincisural fragment with an intact fibularnotch; type 2, the posterolateral fragment extending into the fibular notch; type 3, the posteromedial two-part fragment involving the medial malleolus; type 4, the large posterolateral triangular fragment (involving more than one-third of the notch); and type 5, the nonclassified, irregular, osteoporotic fragments.

**Fig. 1:**
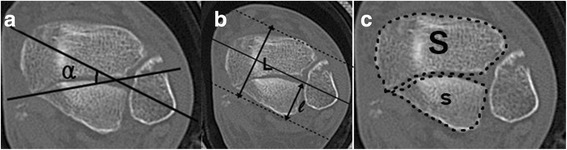
The radiographicmeasurements on the cross section. **a** The cross angle (α) wasdetermined by measuring theangle between the bimalleolaraxis and the major fracture line. **b** The fragment lengthratio (FLR) was determined by calculatingthe percent of length (l)/length (L). **c** The fragment arearatio (FAR) was determined bycalculating the percent of area(s)/area (s + S)

To assess the morphological characteristics of the PMF using CT images, five measurements, which have been used in previous studies [14, 18], were utilized: cross angle (α), fragment length ratio (FLR), fragment area ratio (FAR), sagittal angle (θ), and fragment height (FH). The CT examinations of the ankles were all performed using a conventional 64-channel CT (Toshiba Aquilion TSX-101A 64 channel, Toshiba Medical System, Tokyo, Japan). Axial, sagittal, and coronal (reconstruction thickness of 2 mm) bone window reformation images were used for the measurements. Using the Picture Archiving and Communication System (PACS) system, all measurements were reviewed independently by the two orthopedic surgeons. The cross angle (α, Fig. 1a), FLR (Fig. 1b), and FAR (Fig. 1c) were measured using the axial images. The bimalleolar axis was defined as the center of the fibula and the distal part of the tibia. The cross angle (α) was the angle between the bimalleolar axis and the major fracture line of the PMF on the image at the level of the tibial plafond. The FLR was defined as the ratio between the length of the fragment and the capital diameter of the tibial plafond on the image at the level of the tibial plafond. The length of the fragment and the capital diameter of the tibial plafond were measured by moving the bimalleolar axis parallel from the posterior tibial lip to the anterior tibial lip. The distance between the apex of the fragment and the posterior tibial lip was defined as the length of the fragment (l). The capital diameter of the tibial plafond (L) was measured in the same manner between the anterior and posterior tibial lips. The FAR was measured using a tool in the PACS system, which was used to measure the posterior fragment area (s) and the remaining cross-sectional area of the tibia (S) at the level of the tibial plafond. We calculated the ratio of the fragment area to the total cross-sectional area of the tibial plafond.

**Fig. 2:**
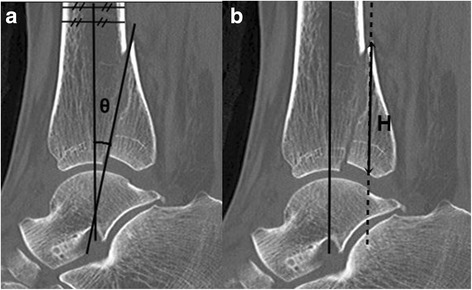
The radiographic measurements on the sagittal reconstructionimages. **a** The sagittal angle (θ) was measured relative to the neutralaxis and the major fracture line of the posterior fragment on thesagittal reconstruction images. **b** The largest distance from the apexof the fragment to the point which is crossed by the dotted line andthe articular surface on consecutive sagittal reconstruction views isdefined as the fragment height (FH)

Using the CT sagittal reconstruction images, we identified a neutral axis (NA) based on the bisection of the midshaft of the tibia. The sagittal angle (θ) (Fig. 2a) was measured relative to the neutral axis and the major fracture line of the posterior fragment on the sagittal reconstruction images. A line parallel to the neutral axis (NA’) was drawn through the apex of the posterior fragment on the sagittal reconstruction views. The FH (Fig. 2b) was defined as the largest distance from the apex of the fragment to the point that was across the joint and the NA’ on the consecutive sagittal reconstruction views. The FH was measured on the sagittal reconstruction views.

### Statistical analysis

Inter- and intraobserver reliabilities were obtained for all radiographic parameters using the intraclass correlation coefficient (ICC). According to the definitions of Landis and Koch [20], ICCs of 0.81–1.00, 0.61–0.80, 0.41–0.60, 0.21–0.40, and 0.00–0.20 were interpreted as excellent, good, moderate, fair, and poor, respectively. A *p* value of < 0.05 was considered statistically significant. The Pearson’s chi-square and Student’s t tests were used to compare sex, age, and radiologic measurements between the SER and PER groups. The Fisher’s exact test was used to compare the morphological difference in PMF morphology of the ankle fractures between the two groups according to the two classification systems. The *p* values on the comparison of the radiologic measurements between the SER and PER groups were calculated using the analysis of covariance adjusted for age and Jan Bartonicek classification.

This study was completed with appropriate institutional review board approval.

## Results

Intraclass correlation coefficients were generated for all radiographic measurements. All measurements were higher than 0.75 (indicating acceptable reliability) and were employed in the study. There was no significant difference in terms of sex between the SER and PER groups, but age was significantly different between these groups. When the two groups were only categorized using the Jan Bartonicek classification, there was a significant difference between the SER and PER groups. The PMF in the SER group mainly had a posterolateral shape, whereas that in the PER group mainly had a posteromedial two-part shape or a large posterolateral triangular shape (*p* = 0.02). These results are shown in Table 1.

**Table 1:** Demographic Data and CategorizingResults using Haraguchi and Jan BartonicekClassification

	SER (*n* = 76)		PER (*n* = 31)		*P*-value^†^
Sex					0.2692
Male	28	(36.8%)	15	(48.4%)	
Female	48	(63.2%)	16	(51.6%)	
Age	49.8	±15.8	38.7	±15.0	0.0011
Haraguchi classification					0.0851
1	58	(76.3%)	18	(58.1%)	
2	17	(22.4%)	13	(41.9%)	
3	1	(1.3%)	0	(0.0%)	
Jan Bartonicek classification					0.0203
1	1	(1.3%)	0	(0.0%)	
2	42	(55.3%)	8	(25.8%)	
3	17	(22.4%)	13	(41.9%)	
4	16	(21.1%)	10	(32.3%)	

Values are number of patients (%) or mean ± SD unless otherwise indicated

*SER* supination-external rotation, *PER* pronation-external rotation

† *P*-values are calculated by Pearson chi-square test, Fisher’s exact test or Student’s t-test as appropriate

The mean (95% CI) and *p* value of the difference between the two groups with regard to radiographic measurements are shown in Table 2. The mean cross angle (α) was not significantly different between the two groups (SER group = 19.4°, PER group = 17.6°). The mean FLR and the mean FH were significantly larger in the PER group than in the SER group (*p* = 0.024, *p* = 0.006). The mean fragment sagittal angle (θ) in the PER group was significantly smaller than that in the SER group (*p* = 0.017).

**Table 2:** Comparison Results ofRadiographic Measurements between SER and PER Groups

	SER (*n* = 76)			PER (*n* = 31)			*P*-value^†^
Alphaangle	13.1	(8.9	17.4)	13.6	(8.6	18.7)	0.7869
FLR	24.7	(21.0	28.4)	28.3	(24.0	32.7)	0.0249
FAR	12.2	(8.8	15.7)	15.0	(10.9	19.1)	0.0762
Fagittal angle	21.2	(17.7	24.7)	17.5	(13.4	21.7)	0.0173
Fragment height	17.6	(14.4	20.9)	21.6	(17.7	25.5)	0.0062

Values are mean (95% CI) adjusted by age and Jan Bartonicek classification

*SER* supination-external rotation, *PER* pronation-external rotation, *FLR* fragment length ratio, *FAR* fragment area ratio

† *P*-values are calculated by analysis of covariance adjusted for age and Jan Bartonicek classification

## Discussion

The PMF is a lesion involving the posterior tibial plafond, including extra-articular osseous avulsions, posterolateral triangular fragment, and impaction of the entire posterior lip [11]. These fragments can commonly occur in various injury patterns, such as ankle fractures, tibia pilon fractures, and tibial spiral shaft fractures [18]. It has been reported that ankle fractures with PMF have a less satisfactory clinical outcome compared to uni- and bimalleolar ankle fractures [3]. Nevertheless, the optimal treatment algorithm of posterior malleolar fractures remains controversial. In recent years, there has been increasing interest on the morphological classification of these injuries and consensus for surgical fixation. Although two classification systems [14, 17] associated with PMF in ankle fractures were reported, both classifications could not distinguish the morphological differences of the PMF according to the injury mechanism of the ankle fracture. Our study aimed at providing the morphological characteristics of the PMF according to the ankle fracture pattern described in the Lauge-Hansen classification, which is useful in determining the type of fixation method to be used.

Traditionally, the lateral view of a plain radiograph has been used to identify the PMF in ankle fractures. However, it has been reported that plain radiographic assessment of the PMF is poorly reliable and accurate although the PMF’s size involving tibial articular surface can be estimated [11, 13]. Accordingly, CT should be required to accurately assess the size and shape of the PMF. In accordance with other studies [14, 17, 18, 21], the authors in the present study used preoperative CT to evaluate the morphology of the PMF, including the fragment size, degree of articular involvement, and FH.

In ankle fractures, Haraguchi et al. [14] were the first to classify 57 cases of PMFs into three types: type I, the posterolateral-oblique type (67%); type II, the medial-extension type (19%); and type III, the small-shell type (14%). However, this classification was based on the fragment size using only a transverse CT scan. Through the investigation performed using CT-based PMF mapping in a series of 45 patients, Mangnus et al. [21] suggested that the morphology of the PMF might be more important than the fragment size alone in making clinical decisions, and they identified Haraguchi type II as a separate fracture pattern. Bartonicek et al. [17] proposed an alternative classification with five categories; the authors argued that a part of type II (the medial-extension type by Haraguchi et al. [14] would be considered a tibial pilon fracture. Moreover, some authors [15, 22] addressed the term posterior pilon variant; this fracture is characterized by a posterior malleolar fracture with posteromedial fragment, such as the medial-extension type II described by Haraguchi et al. [14]. The results of our present study showed that the medial-extension type was more common in the PER group than in the SER group, although the difference was not statistically significant. Bartonicek et al. [17] suggested that the type 3 cases (the posteromedial two-part fragment involving the medial malleolus) could have been caused by the combination of compression force and avulsion, and the type 4 cases (the large posterolateral triangular fragment involving more than one-third of the notch) were probably caused by compression force or as part of a tibia pilon fracture variant. In the present study, when two groups were categorized according to the Jan Bartonicek classification, types 3 and 4 were more statistically and significantly frequent in the PER group than in the SER group. Therefore, this can indicate that the PMF morphological classification presented previously may differ according to the classification of ankle fracture based on the injury mechanism.

Our study showed that the mean FLR and FH were significantly larger in the PER group than in the SER group. Also, the mean fragment sagittal angle (θ) in the PER group was significantly smaller than that in the SER group (*p* = 0.017). In a previous study [18], the area of articular involvement for tibia plafond was significantly associated with FLR. In addition, the fragment sagittal angle (θ) was considered as the vertical nature of the PMF, which can be affected by the shearing force transmitted by the loading. When interpreting our results with reference to the previous study, it can be assumed that the volume of the PMF was larger in the PER group than in the SER group and that the PMF of the PER group has a more vertical nature than that of the SER group.

Knowing the precise mechanism of ankle fractures is important for surgeons to be able to accurately assess the fracture pattern. The Lauge-Hansen classification describes, firstly, the position of the foot at the time of injury and, secondly, the deforming force on the ankle [23]. The ankle may be in one of following positions at the time of trauma: pronation (eversion) and supination (inversion). Three deforming forces may occur: abduction, adduction, and external rotation, which determine the following mechanisms of injury: pronation-abduction (PA), PER, supination-adduction (SA), and SER. The posterior malleolar fracture may occur in both PER and SER injury mechanisms. In stage IV of the PER injury mechanism, posterior malleolar and fibular fracture may occur with or without medial malleolar fracture. However, posterior malleolar and fibular fracture may occur without medial malleolar fracture in stage III of the SER injury mechanism or trimalleolar fracture may occur in stage IV of the SER injury mechanism. In the present study, we compared the PMF morphology of the trimalleolar fracture in stage IV of both the SER and PER injury mechanisms to minimize the bias, which was achieved by adjusting the degree of the deforming force causing the fracture and the type of fracture as equally as possible.

**Fig. 3:**
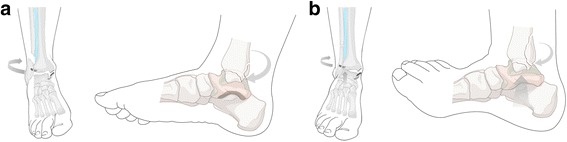
**a** The occurrence of posteriormalleolar fractures in the supination-external rotation stage IV injury is mainly caused by the rotational force. **b** The posterior malleolar fracture in the pronation-external rotationstage IV injury may have occurreddue to a combination of rotational and axial forces

The differences in the PMF morphological characteristics according to the ankle fracture pattern are presumed to be caused by the different foot positions at the time of trauma. In the sequence of supination injuries, fracture of the posterior malleolus may have occurred mainly due to the pulling force of the intact posterior inferior tibiofibular ligament (PITFL) in addition to the rupture of the intact anterior inferior tibiofibular ligament (AITFL) and a fibular spiral fracture. The occurrence of posterior malleolar fractures in the SER injury is mainly caused by the transverse plane rotation. On the other hand, the posterior malleolar fracture in the sequence of pronation injuries may have occurred due to the pushing force of the foot combined with the pulling force in addition to the medial malleolar fracture, involvement of the AITFL with extension into the interosseous membrane, and a spiral or oblique high fibular fracture (Fig. 3). This form of pronation injury is affected not only by the pronation force, but also by the posterior displacement stress combined with lateral malleolar displacement [21, 24] and axial compression force by the talus [25]. In this case, the unstable or large displaced posterior malleolar fracture could be presented. Therefore, the differences of morphological characteristics of the PMF by direction force may be clinically relevant to the different methods for fixation of the PMF. The treatment strategy proposed by the authors is that the smaller fragment due to the rotational force is enough to position the screw, while the larger fragment due to a combination of rotational and axial forces require direct open reduction and internal fixation.

This study has some limitations. The number of patients in the SER group was more than double of that in the PER group. Supination external rotation type has been reported as the most common mechanism of fracture, accounting for 40–70% of all ankle fractures [26]. For this reason, it is presumed that the difference in the number of patients was shown by the two groups. On the other hand, statistically, a compromise power analysis could be performed to determine the appropriate sample size for this study. The control group showed a power (1-β err prob) of 0.845 for detecting a non-centrality parameter δ of 2.03 at a critical t level of 1.019. Thus, this study has a statistically meaningless difference in the number of samples between the two groups, but this difference could lead to reader confusion. The radiographic measurement of the small fragment may have poor precision. Moreover, the definition that distinguishes ankle fractures involving the tibial plafond and tibial pilon fractures is not yet clear. These factors may increase the systematic bias in the present research. Furthermore, the assessment based on CT may be somewhat insufficient to quantify the morphological characteristics of the PMF. In the future, quantification of three-dimensional CT is required to support the results presented in this study.

## Conclusions

The morphological characteristics of the PMF in trimalleolar fracture may differ according to the ankle fracture pattern based on the injury mechanism. With regard to the articular involvement, volume, and vertical nature, the SER-type fracture tends to have a smaller fragment due to the rotational force, whereas the PER-type fracture tends to have a larger fragment due to a combination of rotational and axial forces.

## Abbreviations

AITFL: Anterior inferior tibiofibular ligament
CT: Computed tomography
FAR: Fragment area ratio
FH: Fragment height
FLR: Fragment length ratio
ICC: Intraclass correlation coefficient
PA: Pronation-abduction
PACS: picture archiving and communication system
PER: Pronation-external rotation
PITFL: Posterior inferior tibiofibular ligament
PMF: Posterior malleolar fragment
SA: Supination-adduction
SER: Supination-external rotation
